# Supplementary material for: Assessing Amounts of Genetic Variability in Key Horticultural Traits Underlying Core Korean Breeding Lines of Cut Chrysanthemums
Source: Plants (Basel). 2024 Feb 20;13(5):577. doi: 10.3390/plants13050577 (PMC10934664; doi:10.3390/plants13050577)
Supplement: Supplementary file 1 [file plants-13-00577-s001.zip › plants-2811123-supplementary/Figure S1-9.pptx]

## Slide 1
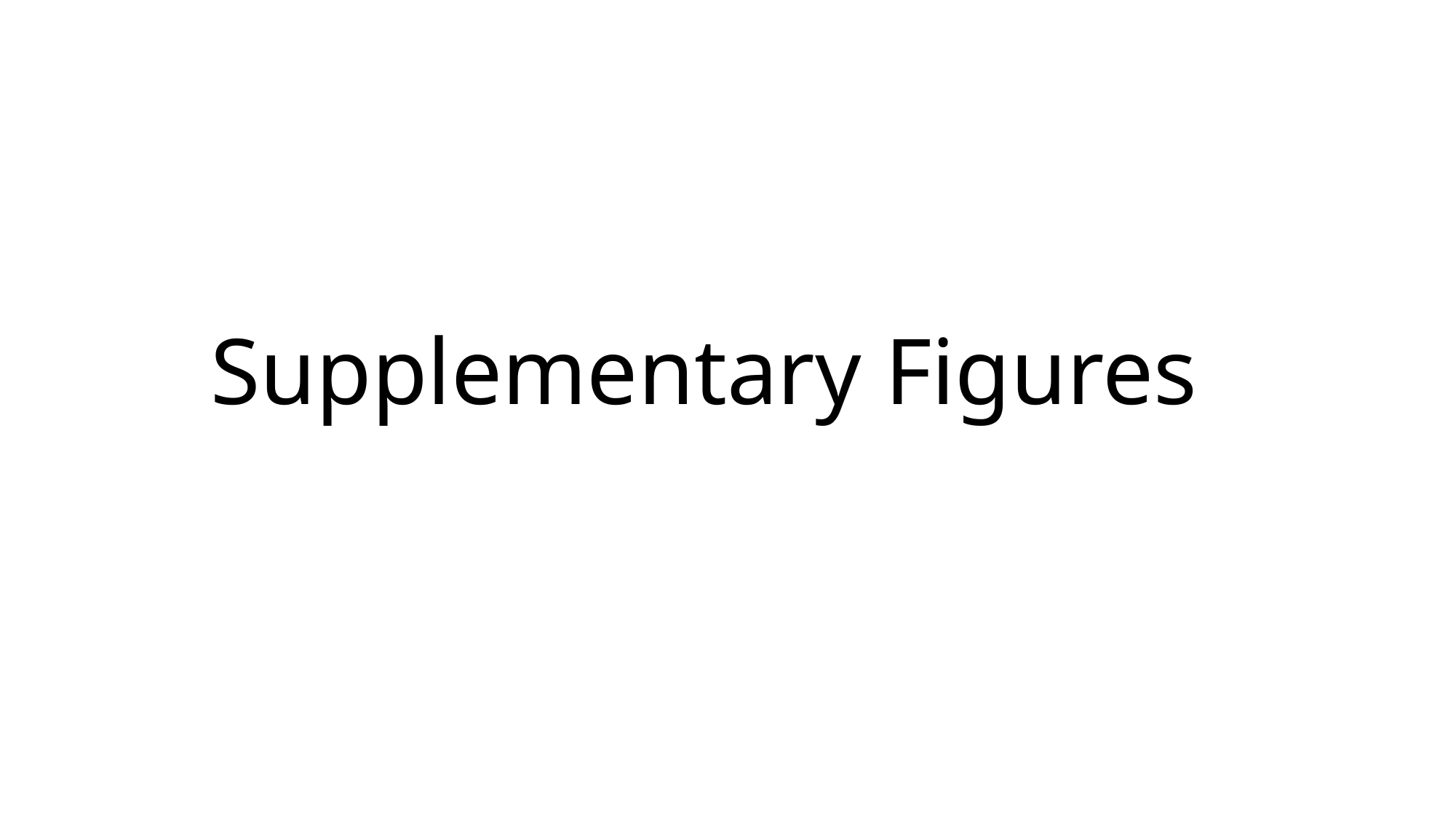

# Supplementary Figures

## Slide 2
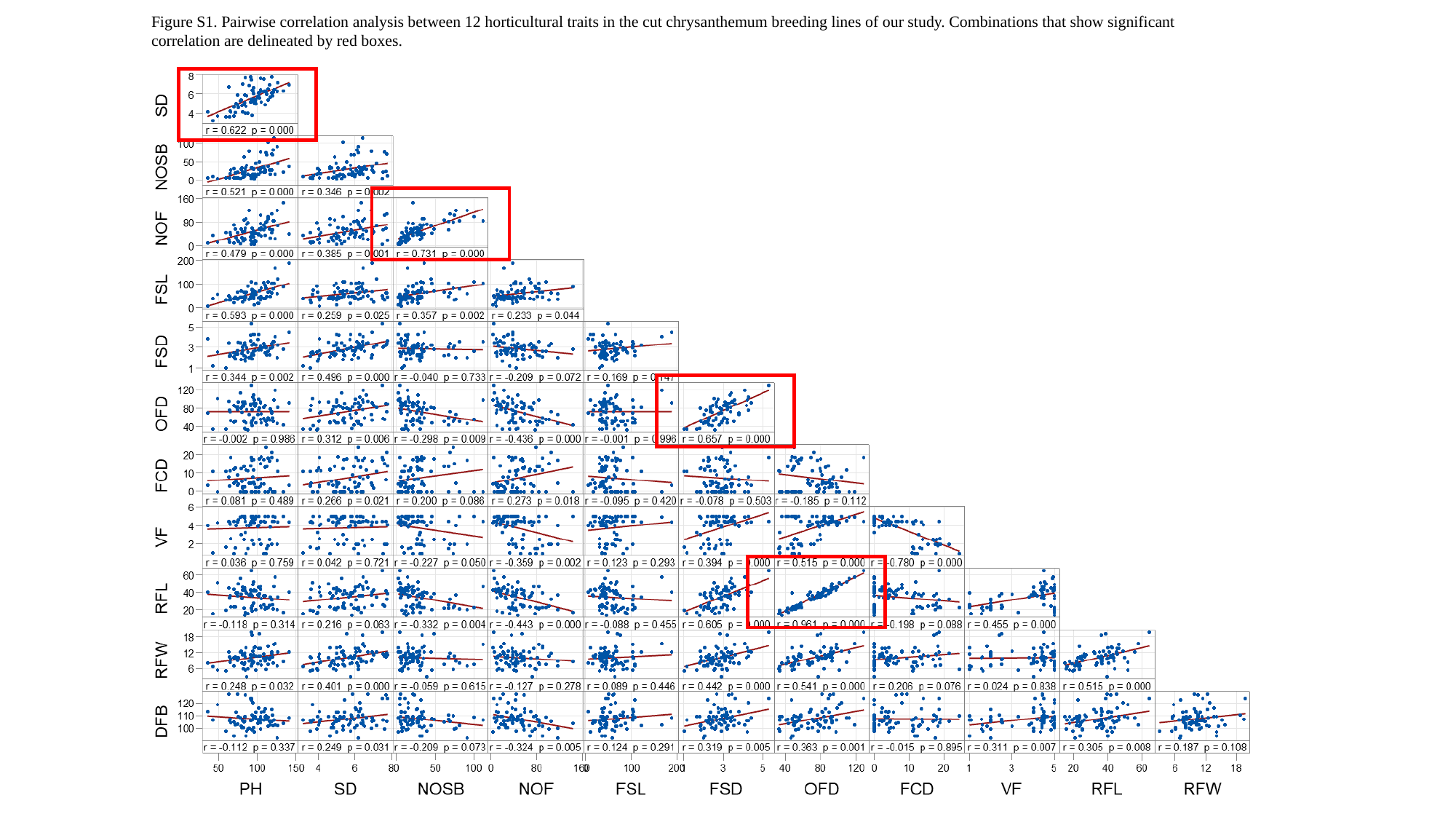

Figure S1. Pairwise correlation analysis between 12 horticultural traits in the cut chrysanthemum breeding lines of our study. Combinations that show significant correlation are delineated by red boxes.

## Slide 3
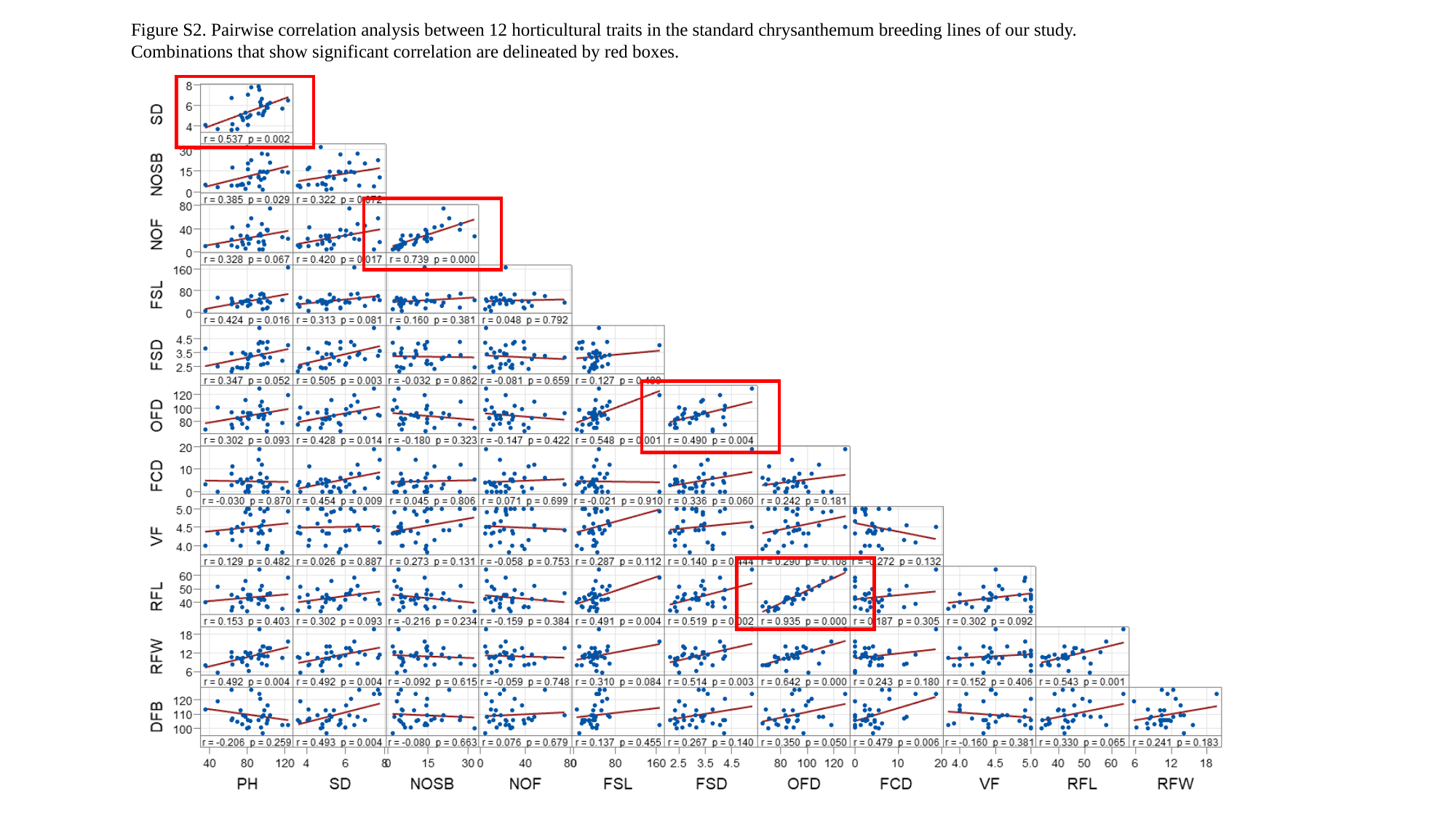

Figure S2. Pairwise correlation analysis between 12 horticultural traits in the standard chrysanthemum breeding lines of our study. Combinations that show significant correlation are delineated by red boxes.

## Slide 4
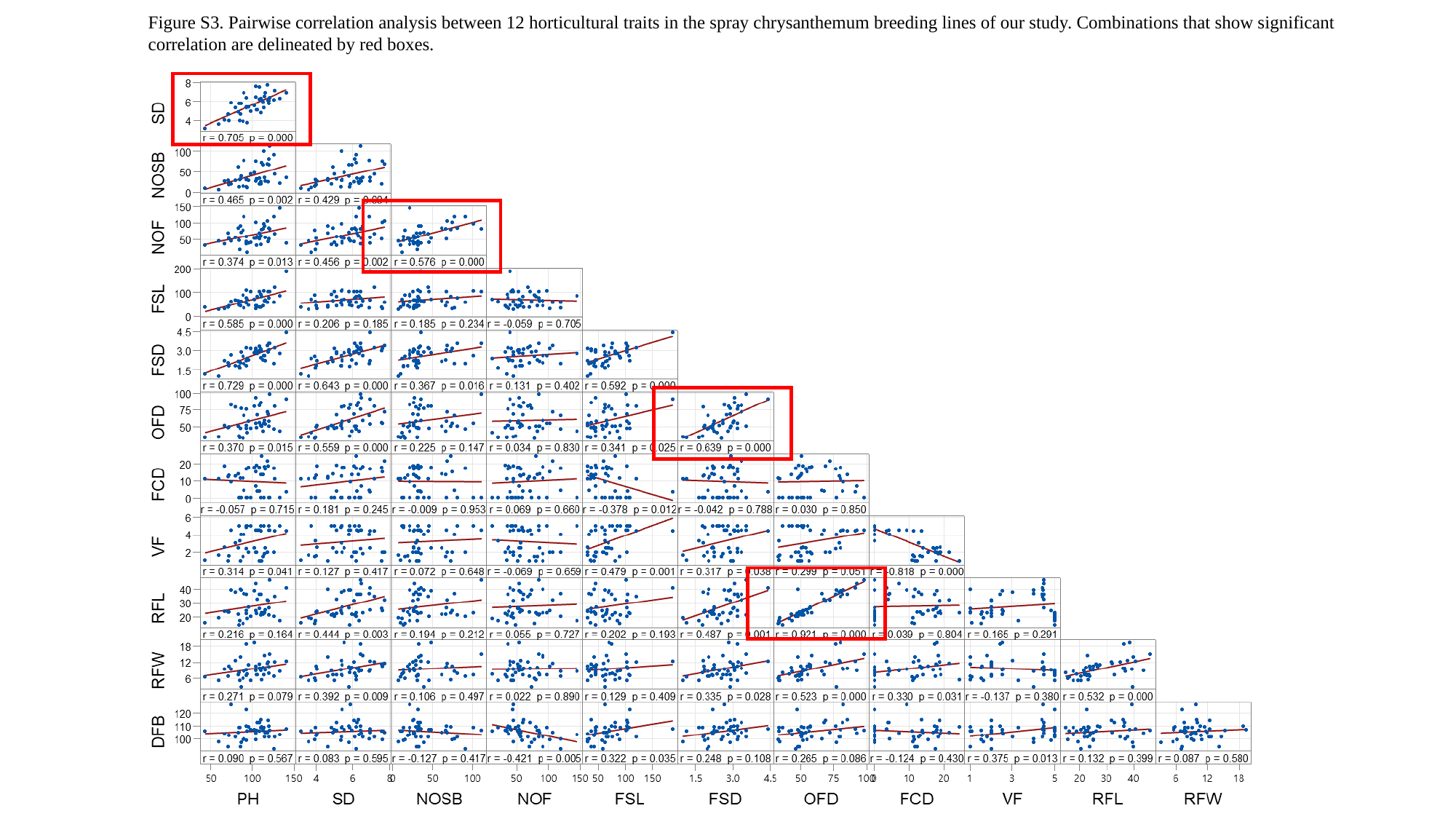

Figure S3. Pairwise correlation analysis between 12 horticultural traits in the spray chrysanthemum breeding lines of our study. Combinations that show significant correlation are delineated by red boxes.

## Slide 5
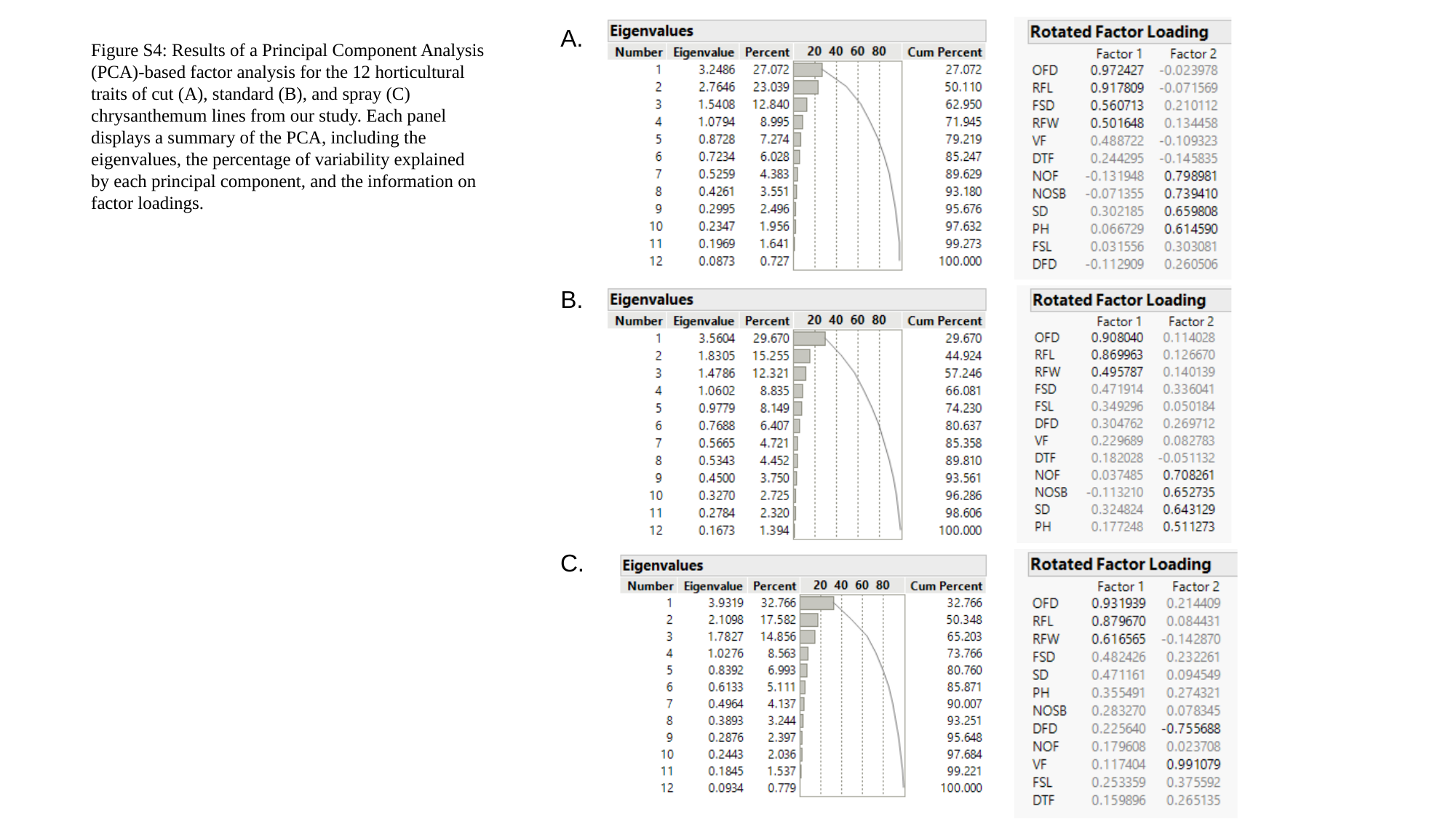

A.
Figure S4: Results of a Principal Component Analysis (PCA)-based factor analysis for the 12 horticultural traits of cut (A), standard (B), and spray (C) chrysanthemum lines from our study. Each panel displays a summary of the PCA, including the eigenvalues, the percentage of variability explained by each principal component, and the information on factor loadings.
B.
C.

## Slide 6
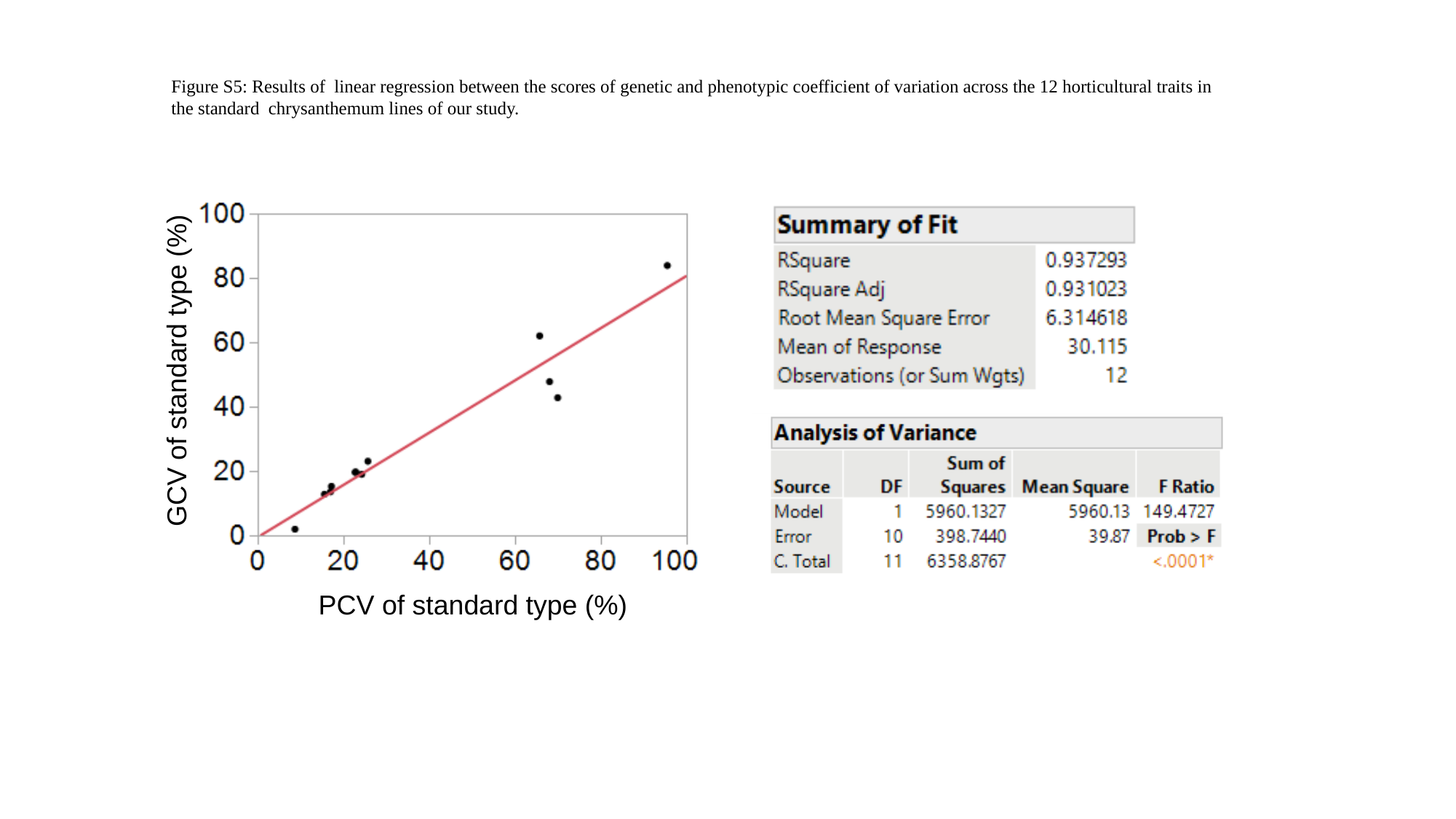

Figure S5: Results of linear regression between the scores of genetic and phenotypic coefficient of variation across the 12 horticultural traits in the standard chrysanthemum lines of our study.
GCV of standard type (%)
PCV of standard type (%)

## Slide 7
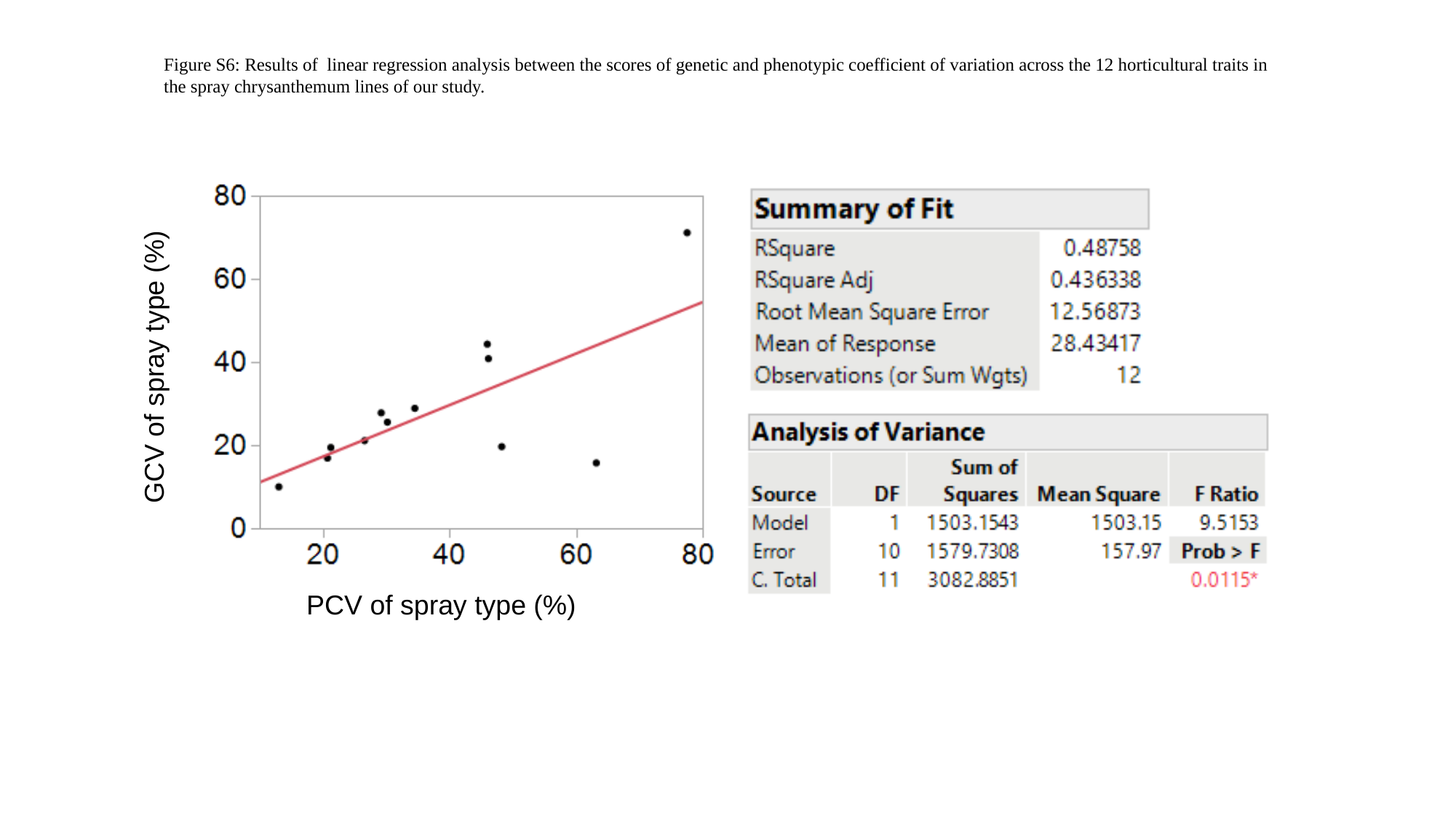

Figure S6: Results of linear regression analysis between the scores of genetic and phenotypic coefficient of variation across the 12 horticultural traits in the spray chrysanthemum lines of our study.
GCV of spray type (%)
PCV of spray type (%)

## Slide 8
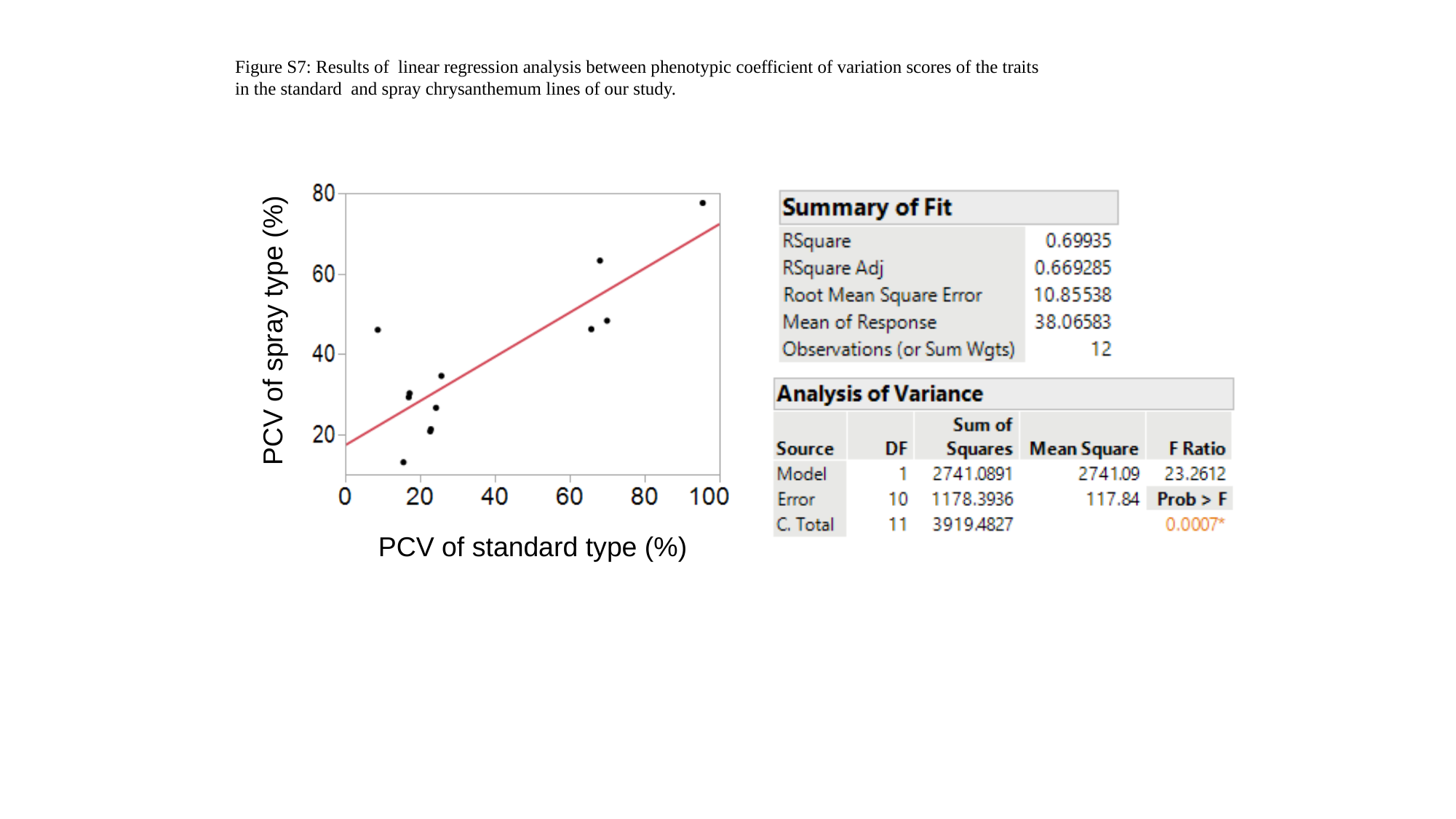

Figure S7: Results of linear regression analysis between phenotypic coefficient of variation scores of the traits in the standard and spray chrysanthemum lines of our study.
PCV of spray type (%)
PCV of standard type (%)

## Slide 9
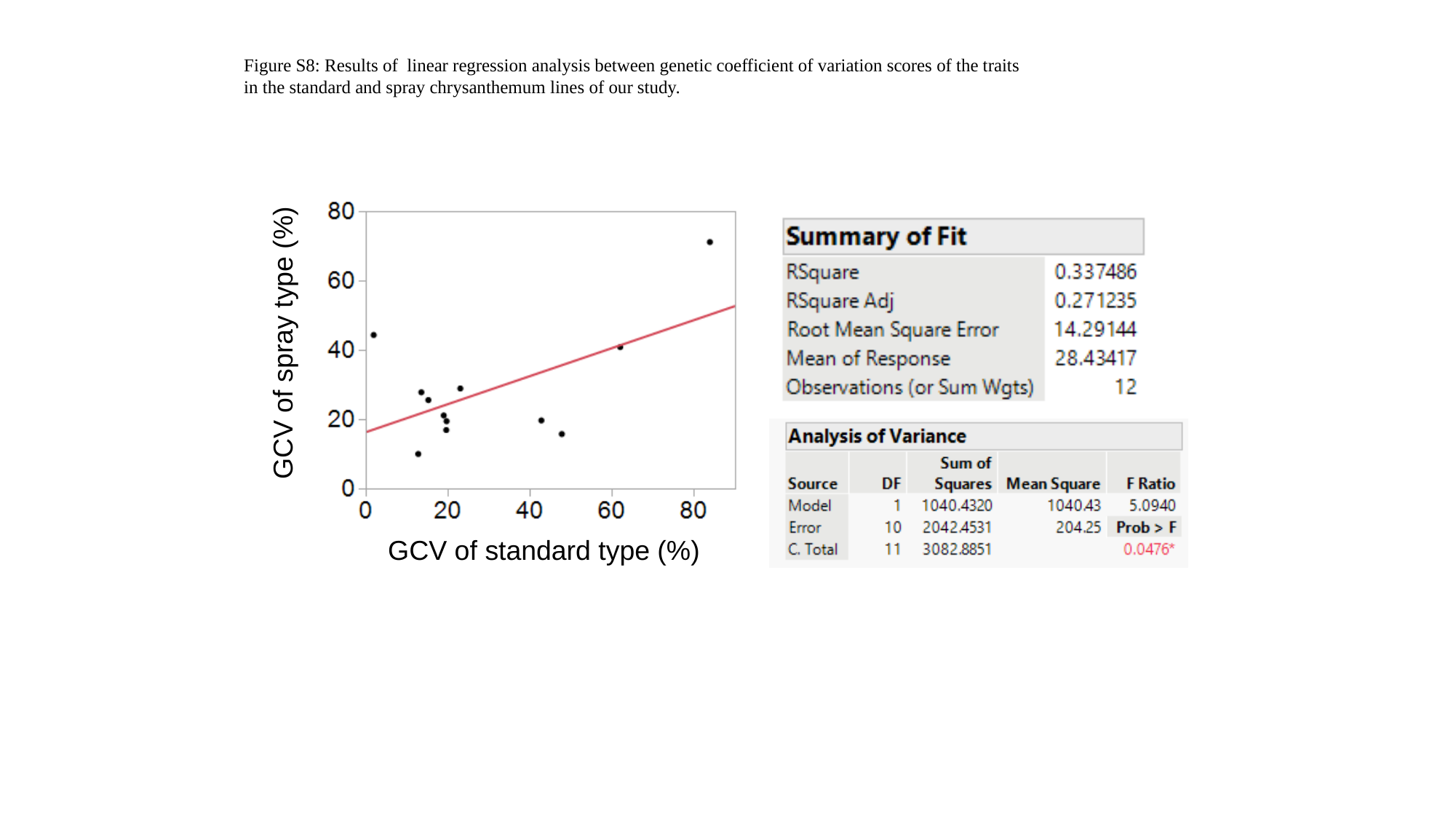

Figure S8: Results of linear regression analysis between genetic coefficient of variation scores of the traits in the standard and spray chrysanthemum lines of our study.
GCV of spray type (%)
GCV of standard type (%)

## Slide 10
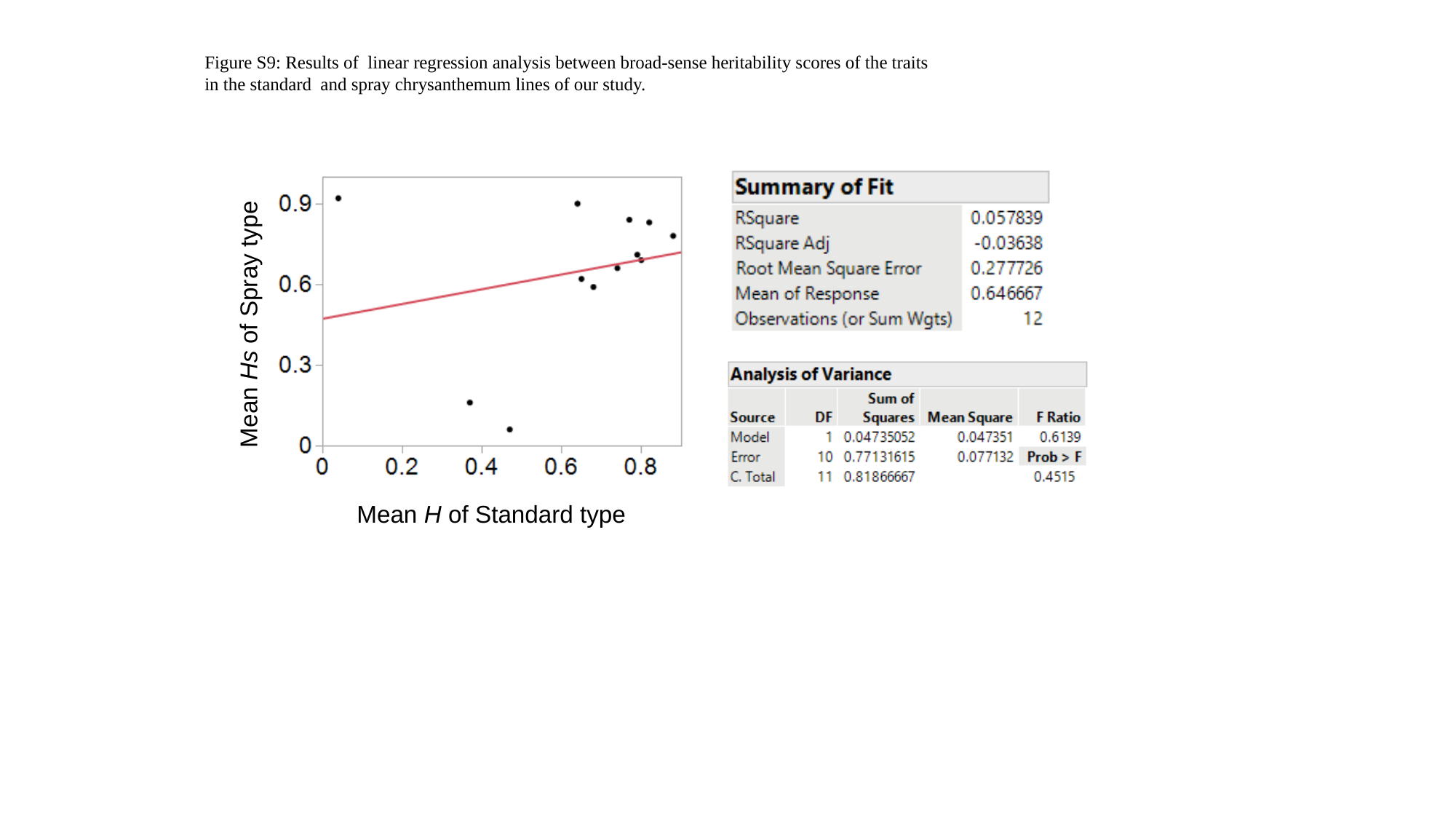

Figure S9: Results of linear regression analysis between broad-sense heritability scores of the traits in the standard and spray chrysanthemum lines of our study.
Mean Hs of Spray type
Mean H of Standard type
