# Supplementary material for: Assessing Amounts of Genetic Variability in Key Horticultural Traits Underlying Core Korean Breeding Lines of Cut Chrysanthemums
Source: Plants (Basel). 2024 Feb 20;13(5):577. doi: 10.3390/plants13050577 (PMC10934664; doi:10.3390/plants13050577)
Supplement: Supplementary file 1 [file plants-13-00577-s001.zip › plants-2811123-supplementary/Table S1_1.pdf]

Table S1. No. of the cut chrysanthemum lines by various types used in this study.

Chrysanthemum types		No. of lines
Standard		31
Spray	Double	12
	Single	21
	Pompon	11
Total		75
