# Supplementary material for: Assessing Amounts of Genetic Variability in Key Horticultural Traits Underlying Core Korean Breeding Lines of Cut Chrysanthemums
Source: Plants (Basel). 2024 Feb 20;13(5):577. doi: 10.3390/plants13050577 (PMC10934664; doi:10.3390/plants13050577)
Supplement: Supplementary file 1 [file plants-13-00577-s001.zip › plants-2811123-supplementary/Table S2_1.pdf]

Table S2. List of cut chrysanthemum lines used in this study.

No.	Cultivar	Type	Provider
1	Wonkyo-183	Standard	NIHHS[z]
2	Baekgang	Standard	NIHHS
3	Nami	Standard	NIHHS
4	WR3	Standard	NIHHS
5	WR4	Standard	NIHHS
6	WR32	Standard	NIHHS
7	WR33	Standard	NIHHS
8	WR65	Standard	NIHHS
9	WR88	Standard	NIHHS
10	JiB18	Standard	NIHHS
11	Isei	Standard	NIHHS
12	Baegma	Standard	NIHHS
13	13-508	Standard	NIHHS
14	Wonkyo-184	Standard	NIHHS
15	Wonkyo-200	Standard	NIHHS
16	13-003-02	Standard	FRIC_ARES[y]
17	17-152-01	Standard	FRIC_ARES
18	11-048-01	Standard	FRIC_ARES
19	Baegseon1	Standard	FRIC_ARES
20	12-154-01	Standard	FRIC_ARES
21	13-076-01	Standard	FRIC_ARES
22	Purple Beam	Standard	FBRIG_ARES[x]
23	Seol Song	Standard	FBRIG_ARES
24	Seinoise	Standard	FBRIG_ARES
25	Baegseon2	Standard	FBRIG_ARES
26	Yong Ma	Standard	FBRIG_ARES
27	Seon Mi	Standard	FBRIG_ARES
28	Seol Mi	Standard	FBRIG_ARES
29	Su Mi	Standard	FBRIG_ARES
30	Dan Mi	Standard	FBRIG_ARES
31	Magnum	Standard	FBRIG_ARES
32	11-063-01	Spray	FBRIG_ARES
33	SJ47	Spray	NIHHS
34	WR53	Spray	NIHHS
35	WR72	Spray	NIHHS

36	WR98	Spray	NIHHS
37	HJ66	Spray	NIHHS
38	13-055-01	Spray	FRIC_ARES
39	Jembera Lime	Spray	FBRIG_ARES
40	Jembera	Spray	FBRIG_ARES
41	Daiansa	Spray	FBRIG_ARES
42	Baleuka	Spray	FBRIG_ARES
43	Pib	Spray	FBRIG_ARES
44	Green Diamond	Spray	NIHHS
45	Poyeru90	Spray	FRIC_ARES
46	SJc1	Spray	NIHHS
47	Field Green	Spray	NIHHS
48	Manito	Spray	NIHHS
49	Cherry Blossom	Spray	NIHHS
50	Glory Pink	Spray	NIHHS
51	Red Marble	Spray	NIHHS
52	18-36	Spray	NIHHS
53	11-120	Spray	NIHHS
54	Ayano	Spray	FRIC_ARES
55	Hwahwa	Spray	FRIC_ARES
56	Seogyu	Spray	FRIC_ARES
57	Queen star	Spray	FRIC_ARES
58	Cheonglyu	Spray	FRIC_ARES
59	Lovely salmon	Spray	FRIC_ARES
60	Dancing queen	Spray	FRIC_ARES
61	Perfect	Spray	FRIC_ARES
62	Yes ruby	Spray	FRIC_ARES
63	19-138-02	Spray	FRIC_ARES
64	Yes holic	Spray	FRIC_ARES
65	Pink Pangpang	Spray	NIHHS
66	Snow Pangpang	Spray	NIHHS
67	Peach Pangpang	Spray	NIHHS
68	17-283-03	Spray	FRIC_ARES
69	Orang pop	Spray	FBRIG_ARES
70	Sunny pop	Spray	FBRIG_ARES
71	Purple pop	Spray	FBRIG_ARES
72	White pop	Spray	FBRIG_ARES
73	Lime pop	Spray	FBRIG_ARES

74	Pink pop	Spray	FBRIG_ARES
75	Magic pop	Spray	FBRIG_ARES

[z], NIHHS stands for the National Institute of Horticultural and Herbal Science, Korea

[y], FRIC_ARES, Flower Research Institute of Chungcheongnam-do Agricultural Research & Extension Services, Korea

[x], FBRIG_ARES, Flower Breeding Research Institute of Gyeongsangnam-do Agricultural Research & Extension Services, Korea
