# Supplementary material for: Assessing Amounts of Genetic Variability in Key Horticultural Traits Underlying Core Korean Breeding Lines of Cut Chrysanthemums
Source: Plants (Basel). 2024 Feb 20;13(5):577. doi: 10.3390/plants13050577 (PMC10934664; doi:10.3390/plants13050577)
Supplement: Supplementary file 1 [file plants-13-00577-s001.zip › plants-2811123-supplementary/Table S3_1.pdf]

Table S3. List of the methods for the phenotypic assessments of our study.

No	Traits	abbreviation	Method of the phenotypic evaluation
1	Plant height (cm)	PH	The PH is to be measured from the soil line to the top of the first flower.
2	Stem diameter (mm)	SD	The SD is to be measured on the point of the half position of the PH
3	No. of side branches (each)	NOSB	The NOSB is to be counted for the main branch number across the PH.
4	No of flowers (each)	NOF	The NOF is to be counted for the flowers across the PH.
5	Flower stem length (mm)	FSL	The FSL is to be measured for the flower stem length on the first flower.
6	Flower stem diameter (mm)	FSD	The FSD is to be measured for the flower stem diameter on the first flower.
7	overall flower diameter (mm)	OFD	The OFD is to be measured for the diameter of the first flower.
8	Disk flower diameter (mm)	DFD	The DFD is to be measured for the diameter of the disk flower on the first flower.
9	Volume of flower (1~6 scale)	VF	The VF is to be evaluated on the volume of flower for the first flower with 1-5 scale.
10	Ray flower length (mm)	RFL	The RFL is to be measured for the average length of the ray flower (n=3) on the outer side of the first flower.
11	Ray flower width (mm)	RFW	The RFW is to be measured for the average width of the ray flower (n=3) on the outer side of the first flower.
12	Days to flowering (days)	DTF	The DTF is to be measured for the days from the planting, requiring for 50% of blooming of each chrysanthemum line.
