# Supplementary material for: Assessing Amounts of Genetic Variability in Key Horticultural Traits Underlying Core Korean Breeding Lines of Cut Chrysanthemums
Source: Plants (Basel). 2024 Feb 20;13(5):577. doi: 10.3390/plants13050577 (PMC10934664; doi:10.3390/plants13050577)
Supplement: Supplementary file 1 [file plants-13-00577-s001.zip › plants-2811123-supplementary/Table S4_1.pdf]

Table S4. Descriptive statistics of the 12 horticultural traits among the standard chrysanthemum breeding lines.

Variable(trait)	Mean	SE Mean	Range	Q1	Median	Q3
PH	85.45	1.14	20~142	74	86	100
SD	5.429	0.0872	1.78~12.02	4.3025	5.3	6.3575
NOSB	11.852	0.601	0~71	4	8	16
NOF	24.63	1.41	1~160	7	15	31
FSL	42.57	1.74	1~294	25	39	53
FSD	3.2205	0.057	0.77~6.45	2.4675	3.17	3.8925
OFD	89.57	1.11	10~172	75	90	105
DFD	4.466	0.283	0~23	0	3	7
VF	4.5131	0.0325	3~5	4	5	5
RFL	43.77	0.524	23~75	36	42	50
RFW	10.945	0.18	4~26	9	11	12
DTF	109.05	0.554	88~159	101	107	113

s
