# Supplementary material for: Assessing Amounts of Genetic Variability in Key Horticultural Traits Underlying Core Korean Breeding Lines of Cut Chrysanthemums
Source: Plants (Basel). 2024 Feb 20;13(5):577. doi: 10.3390/plants13050577 (PMC10934664; doi:10.3390/plants13050577)
Supplement: Supplementary file 1 [file plants-13-00577-s001.zip › plants-2811123-supplementary/Table S5_1_1.pdf]

Table S5. Descriptive statistics of the 12 horticultural traits among the spray chrysanthemum breeding lines.

Variable	Flower type	Mean	SE Mean	Minimum	Q1	Median	Q3
PH	Overall	100.2	1.1	33~160	84.0	104.0	118.0
SD	Overall	5.7	0.1	1.28~10.83	4.5	5.6	6.9
NOSB	Overall	40.6	2.2	2~250	13.0	24.0	44.0
NOF	Overall	64.5	2.6	2~320	24.0	47.0	86.0
FSL	Overall	69.9	1.8	2~290	44.8	62.0	92.0
FSD	Overall	2.6	0.0	0.33~5.24	2.0	2.6	3.2
OFD	Overall	59.4	0.9	19~115	45.0	55.0	73.5
DFD	Overall	9.8	0.4	0~37	0.0	10.0	17.0
VF	Overall	3.1	0.1	1~5	2.0	3.0	5.0
RFL	Overall	27.8	0.5	5~70	20.0	25.0	34.5
RFW	Overall	9.8	0.2	1~29	7.0	9.0	12.0
DTF	Overall	106.0	0.4	85~142	100.0	107.0	109.0
